# Persistent gastro-cutaneous fistula closure with tack suture system in the setting of a severe esophageal stricture

**DOI:** 10.1016/j.vgie.2022.02.010

**Published:** 2022-04-07

**Authors:** Mohammad Sultan, Ricardo Romero, John Evans, Janak Shah, Abdul El-Chafic

**Affiliations:** Ochsner Medical Center, New Orleans, Louisiana

**Keywords:** GCF, gastro-cutaneous fistula, OTSC, over-the-scope clip

## Abstract

Video 1 Endoscopic video demonstrating an esophageal stricture, which was dilated, resulting in a deep tear without perforation. Previously placed hemoclips were visualized and removed. argon plasma coagulation was applied to denude the fistula epithelium. Subsequent demonstration of the use of the tack suturing system to close a persistent gastro-cutaneous PEG fistula.

Endoscopic video demonstrating an esophageal stricture, which was dilated, resulting in a deep tear without perforation. Previously placed hemoclips were visualized and removed. argon plasma coagulation was applied to denude the fistula epithelium. Subsequent demonstration of the use of the tack suturing system to close a persistent gastro-cutaneous PEG fistula.

## Case presentation

A 64-year-old man with a history of metastatic squamous cell carcinoma of the larynx underwent laryngopharyngectomy with good response to adjuvant combination of immunotherapy and chemotherapy. Postoperatively, the patient developed symptomatic esophageal stricture requiring serial sessions of endoscopic dilation, which resulted in improved nutritional status and the removal of a previously placed PEG tube. However, a gastro-cutaneous PEG fistula (GCF) persisted 5 months after PEG tube removal, significantly affecting the patient’s quality of life. Prior attempts at endoscopic closure with argon plasma coagulation + endoscopic clips did not successfully close the fistula, and the patient was referred to our service for further management options.

Gastrostomy tracts typically close in 24 to 72 hours after PEG tube removal. Persistent GCF is rare and usually results from prolonged duration of gastrostomy, leading to epithelization of the fistulous tract. Other suggested etiologies of persistent GCF include immunosuppression, stomal infection, increased gastric acidity, delayed gastric emptying, and malnutrition.

The patient’s medical history includes follicular lymphoma, stage 3 chronic kidney disease, hypertension, hypersensitivity lung disease, type 2 diabetes, paroxysmal atrial fibrillation, and alcohol abuse.

## Procedure

**Figure 1:**
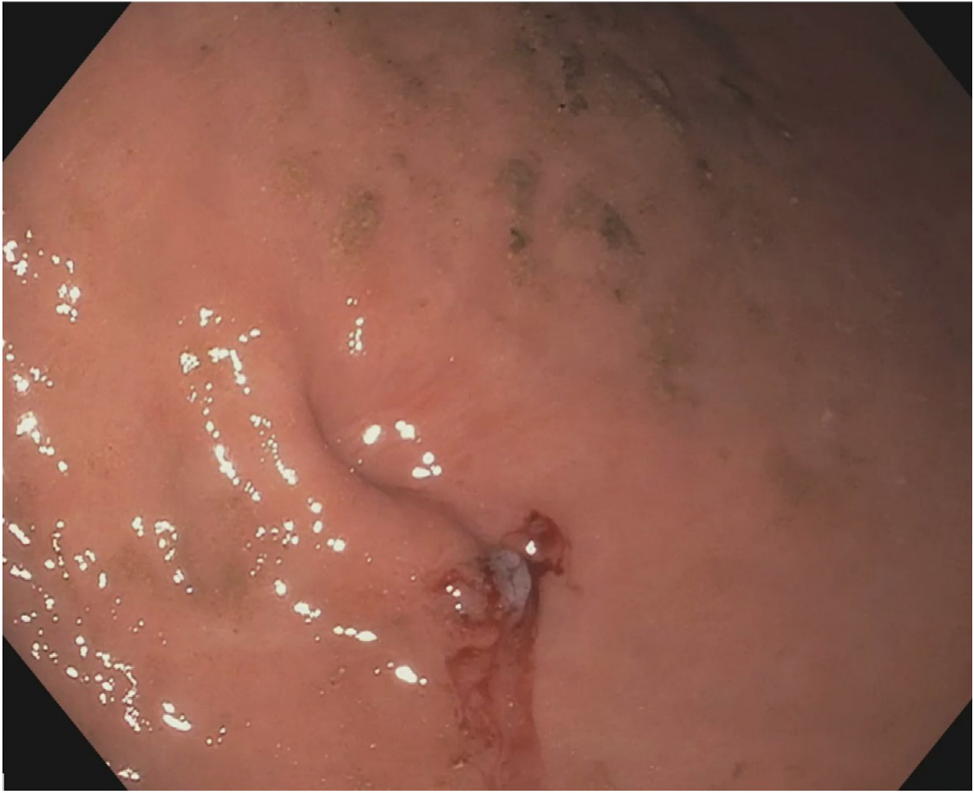
Gastro-cutaneous fistula.

An esophageal anastomotic stricture measuring 7 mm in diameter was encountered in the upper esophagus. A standard gastroscope (9.9 mm outer diameter) could not traverse the stricture. As such, dilation was performed with a controlled radial expansion balloon up to 12 mm. This resulted in a deep tear without perforation. The standard gastroscope was subsequently able to traverse the stricture after dilation, with only mild resistance. The PEG gastro-cutaneous fistula was visualized in the gastric body with the previously placed endoscopic clips lying adjacent to the fistula (Fig. 1).

**Figure 2:**
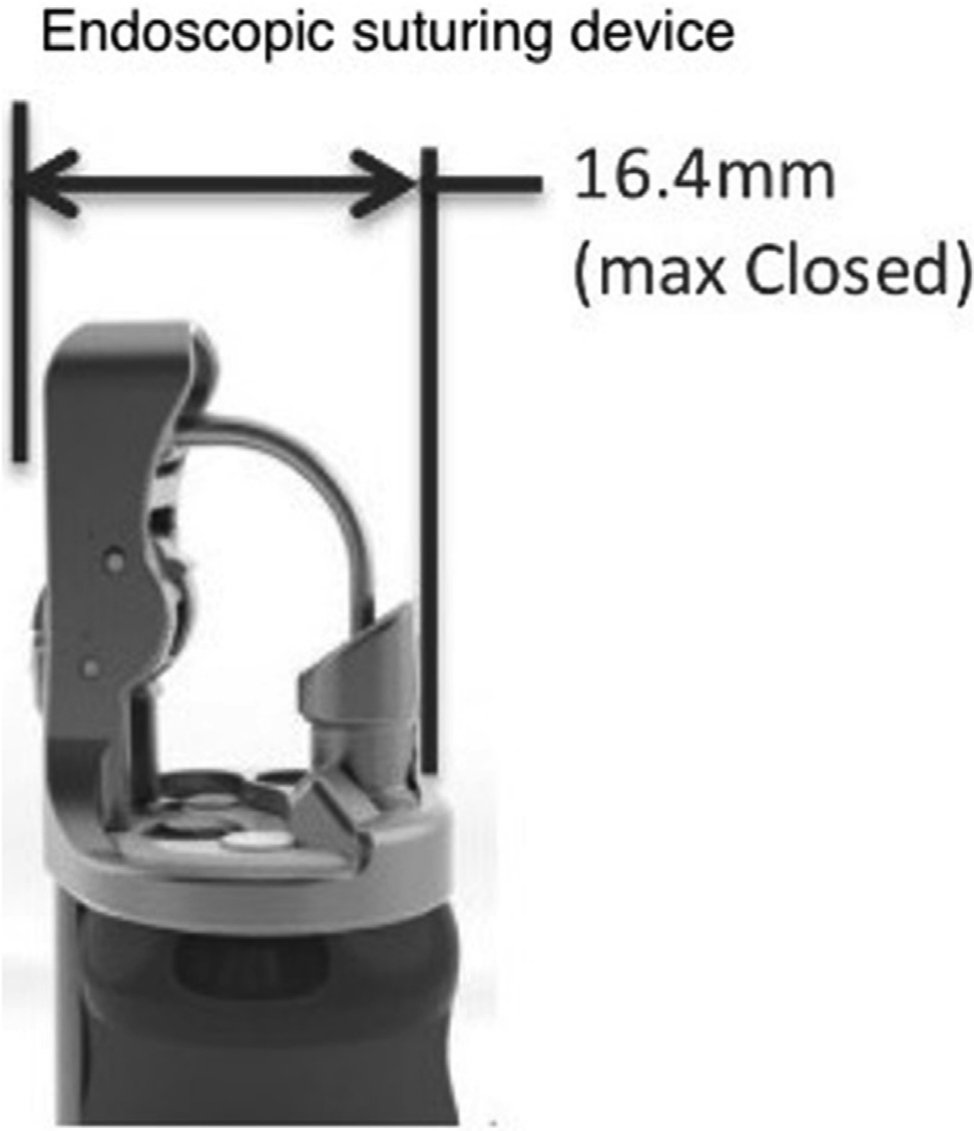
Diameter of endoscopic suturing device.

**Figure 3:**
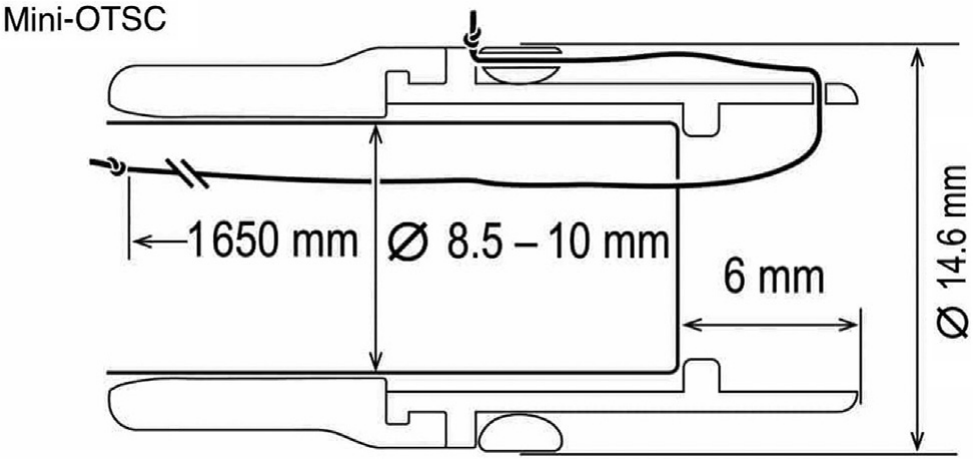
Diameter of the mini over-the-scope clip device.

**Figure 5:**
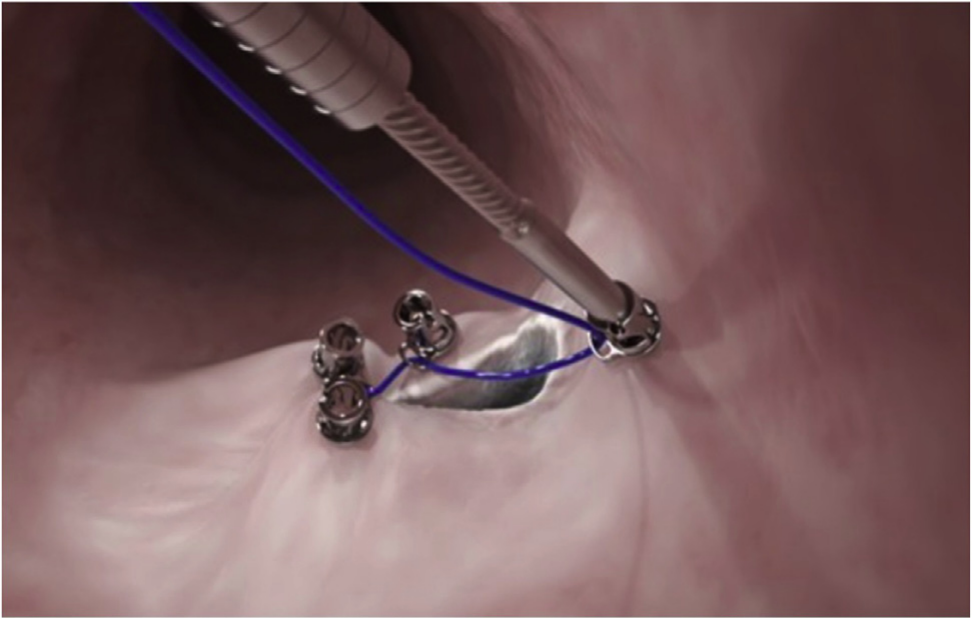
Ex vivo footage of the tack suturing system.

Gastro-cutaneous fistulas that develop at prior PEG sites are often successfully closed via endoscopic suturing or the over-the-scope clip (OTSC). However, the endoscopic suturing device measures 16.4 mm in diameter (Fig. 2) and the mini OTSC device measures 14.6 mm (Fig. 3); neither of these could pass through the stricture in our patient. Notably, a tack suturing system has recently become available as a suture-based, deep submucosal and intramuscular enhanced fixation tool that can be used through the 2.8-mm working channel of a standard gastroscope (Fig. 5). We decided to proceed with this device. The previously placed endoscopic clips were removed. Argon plasma coagulation was then applied to denude the fistula epithelium.

The tacking device was inserted with preloaded helix tack and scope liner into the scope channel. The scope was maneuvered to the target site, and the first tack was placed about 10 mm from the fistula site. The device catheter was removed from the scope, and the handle was reset. The helix tack was reloaded. The scope was maneuvered, and the second site was targeted. The tack was then deployed. This process was repeated until 4 tacks were appropriately positioned. The device catheter and scope liner were then removed from the working channel, leaving the suture in place.

**Figure 4:**
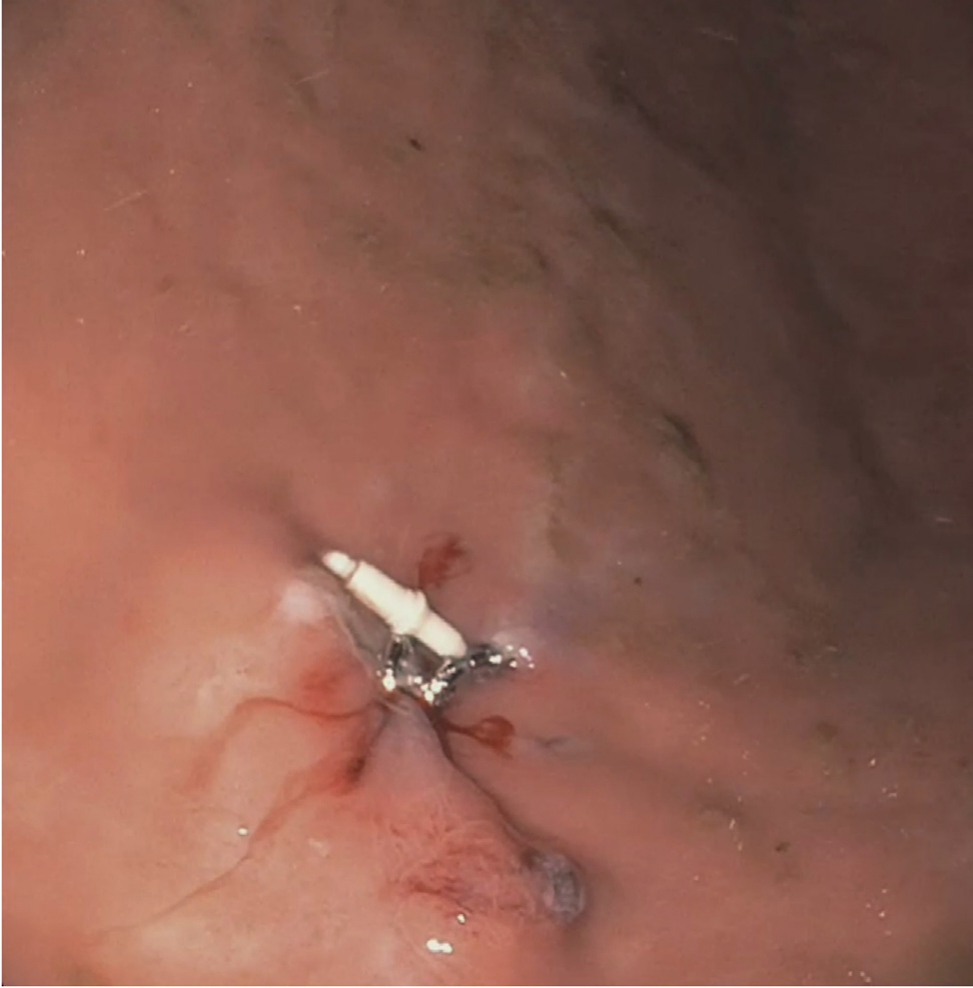
Gastro-cutaneous fistula closed with the tack suturing system.

While holding slight suture tension, we inserted the cinch into the working channel. While holding the cinch catheter in place, we applied tension to the suture until the helix tacks and tissue were appropriately approximated. With slight suture tension, the cinch was then deployed. The remaining cinch and suture were removed from the channel, and the final closure was inspected. The defect appeared to be satisfactorily repaired (Fig. 4). Our patient was followed-up 2 weeks after closure without any further leakage through the fistula (Video 1, available online at www.giejournal.org).

## Conclusions

Our case presents the use of a novel tack suturing system to close a persistent gastro-cutaneous PEG fistula in the setting of a severe esophageal stricture. Commonly used defect closing devices such as the OTSC and endoscopic suturing device were unable to traverse the stricture. The tack suture system is a potentially useful tool when limited to the parameters of a standard gastroscope, as was the case here.

Unfortunately, 3 weeks later, the GCF tract reopened, but with minimal leakage. The patient ultimately had interval staging positron emission tomography scans with evidence of disease progression on chemotherapy and opted to transition to comfort-focused measures. He subsequently enrolled in hospice until he died of his illness. Failure of prolonged fistula closure is likely secondary to the patient’s immunosuppression, disease progression, and malnutrition. We also admit that the new tack suture system is not a full-thickness suturing device and may be inferior to other defect-closing devices.

## Disclosure

*Dr Shah is a consultant for Olympus and BSCI. All other authors disclosed no financial relationships.*

## Supplementary data

Video 1 Endoscopic video demonstrating an esophageal stricture, which was dilated, resulting in a deep tear without perforation. Previously placed hemoclips were visualized and removed. argon plasma coagulation was applied to denude the fistula epithelium. Subsequent demonstration of the use of the tack suturing system to close a persistent gastro-cutaneous PEG fistula.

